# High *Plasmodium falciparum* genetic diversity and temporal stability despite control efforts in high transmission settings along the international border between Zambia and the Democratic Republic of the Congo

**DOI:** 10.1186/s12936-019-3023-4

**Published:** 2019-12-04

**Authors:** Julia C. Pringle, Amy Wesolowski, Sophie Berube, Tamaki Kobayashi, Mary E. Gebhardt, Modest Mulenga, Mike Chaponda, Thierry Bobanga, Jonathan J. Juliano, Steven Meshnick, William J. Moss, Giovanna Carpi, Douglas E. Norris

**Affiliations:** 1 W. Harry Feinstone Department of Molecular Microbiology and Immunology, Johns Hopkins Malaria Research Institute, Johns Hopkins Bloomberg School of Public Health, Baltimore, MD 21205 USA; 2 Department of Epidemiology, Johns Hopkins Bloomberg School of Public Health, Baltimore, MD 21205 USA; 3 Department of Biostatistics, Johns Hopkins Bloomberg School of Public Health, Baltimore, MD 21205 USA; 4 Tropical Diseases Research Centre, Ndola, Zambia; 5 Université Protestante au Congo and University of Kinshasa, Kinshasa, Democratic Republic of the Congo; 6 Division of Infectious Diseases, School of Medicine, University of North Carolina, Chapel Hill, NC 27599 USA; 7 Gillings School of Global Public Health, University of North Carolina, Chapel Hill, NC 27599 USA; 8 Department of Biological Sciences, Purdue University, West Lafayette, IN 47907 USA

**Keywords:** Malaria, Genetic, Border, Diversity, Control, Amplicon deep sequencing, *csp*, *ama1*

## Abstract

**Background:**

While the utility of parasite genotyping for malaria elimination has been extensively documented in low to moderate transmission settings, it has been less well-characterized in holoendemic regions. High malaria burden settings have received renewed attention acknowledging their critical role in malaria elimination. Defining the role for parasite genomics in driving these high burden settings towards elimination will enhance future control programme planning.

**Methods:**

Amplicon deep sequencing was used to characterize parasite population genetic diversity at polymorphic *Plasmodium falciparum* loci, *Pfama1* and *Pfcsp*, at two timepoints in June–July 2016 and January–March 2017 in a high transmission region along the international border between Luapula Province, Zambia and Haut-Katanga Province, the Democratic Republic of the Congo (DRC).

**Results:**

High genetic diversity was observed across both seasons and in both countries. No evidence of population structure was observed between parasite populations on either side of the border, suggesting that this region may be one contiguous transmission zone. Despite a decline in parasite prevalence at the sampling locations in Haut-Katanga Province, no genetic signatures of a population bottleneck were detected, suggesting that larger declines in transmission may be required to reduce parasite genetic diversity. Analysing rare variants may be a suitable alternative approach for detecting epidemiologically important genetic signatures in highly diverse populations; however, the challenge is distinguishing true signals from potential artifacts introduced by small sample sizes.

**Conclusions:**

Continuing to explore and document the utility of various parasite genotyping approaches for understanding malaria transmission in holoendemic settings will be valuable to future control and elimination programmes, empowering evidence-based selection of tools and methods to address pertinent questions, thus enabling more efficient resource allocation.

## Background

Significant progress has been made in reducing *Plasmodium falciparum* malaria transmission since the early 2000s, due in part to massive distributions of insecticide-treated bed nets (ITNs), increased coverage with indoor residual spraying (IRS), and the introduction of artemisinin-based combination therapy (ACT) [1, 2]. In fact, it is estimated that the global burden of malaria declined by 40% between 2000 and 2015, leading 35 countries to establish malaria elimination targets as of September 2015 [1, 3]. While the current arsenal of malaria control tools has been broadly effective, it has not been sufficient to reduce transmission everywhere [4]. Despite malaria control programmes, the ten countries with the highest malaria burden in Africa experienced increases in malaria cases between 2015 and 2017 [5, 6]. Acknowledging that the success of malaria control in these and other high burden regions is critical for the attainment of malaria elimination targets, the World Health Organization (WHO) renewed its focus on high transmission settings as a key component of malaria elimination programmes in the recent High Burden to High Impact (HBHI) response plan [6]. Achieving malaria control in regions where transmission has previously been refractory to interventions will require an enhanced understanding of the unique mechanisms that perpetuate transmission in different settings and data-driven approaches that target them [6].

*Plasmodium falciparum* molecular epidemiology has emerged as a tool to genetically track transmission patterns [7–10]. Population genetic diversity and multiplicity of infection (MOI) have been used to monitor changes in transmission intensity and evaluate whether programmes or interventions have altered transmission patterns [11–19]. Parasite genotyping may allow more refined discrimination of parasite locality of origin than travel surveys alone, possibly enabling gene flow between populations to be estimated [20–22] and source populations of on-going transmission to be identified [23]. Parasite genetic approaches have demonstrated their value in complementing routine epidemiological and entomological surveillance to inform control programmes in moderate to low transmission settings where elimination efforts have been concentrated [7]. With renewed attention to malaria control in high burden regions as a component of elimination planning, it is important to define the potential role of molecular epidemiology in such settings.

Zambia, a malaria-endemic country in southern Africa, aims to eliminate malaria from every district by 2021. As a country, Zambia has made great strides towards achieving this goal. In fact, *P. falciparum* prevalence by microscopy among children under 5 years old declined from 19.4% in 2015 to 9.1% in 2018 [24]. Unfortunately, the success in malaria control has been heterogeneous across the country. Luapula Province, located in northern Zambia along the international border with the Democratic Republic of the Congo (DRC), continues to experience holoendemic transmission despite use of control interventions for over a decade [4]. In Nchelenge District, located in Luapula Province, ITN distributions were carried out in 2007, 2011, and 2014, and IRS has been conducted annually, using pyrethroids between 2008 and 2010, carbamates between 2011 and 2012, and the organophosphate, pirimiphos-methyl (Actellic^®^ 300CS), since 2014 [4, 25]. IRS with Actellic^®^ led to a moderate decrease in *P. falciparum* prevalence by 25% in the sprayed regions within Nchelenge District; however, the effect was short-lived, lasting only for 6 months after spraying, and did not impact unsprayed areas [25]. Despite control measures, Luapula Province, including Nchelenge District, continues to experience the highest malaria prevalence among children under 5 years in the country at 30.4% by RDT [24, 26].

Across Lake Mweru from Nchelenge, situated along the international border, are two villages, Kilwa and Kashobwe in Haut-Katanga Province, the DRC. The most recent Demographic and Health Survey (DHS) from the DRC conducted in 2013–2014 reported that 23.1% of children ages 6 months to 5 years tested positive for malaria by microscopy in Haut-Katanga Province [27]. This Province has received fewer malaria control interventions historically compared to neighboring Nchelenge District in Zambia. Four cross-sectional surveys in Kilwa and Kashobwe conducted between August 2016 and July 2017 revealed that very few households (range: 0.2–2.5%) reported ever receiving IRS in these villages (Kobayashi, unpublished). During September and October 2016, a large-scale ITN distribution took place in Haut-Katanga Province. Epidemiological survey data revealed that following the ITN campaign, there was a significant increase in the proportion of individuals who reported sleeping under a bed net and a decline in parasite prevalence by microscopy in Kilwa and Kashobwe from 32% in 2016 to 18% in 2017 (Kobayashi, unpublished).

In high transmission settings including Nchelenge District, Zambia and Kilwa and Kashobwe, the DRC, individuals are typically infected with multiple genetically distinct parasites. Classic population genetic approaches that utilize neutrally-evolving SNPs or microsatellites across the genome inadequately address high polyclonality. Amplicon deep sequencing enables each parasite clone in an infection to be enumerated, but typically only at specific, high-diversity and non-neutral amplicons in the genome. Given the interest in molecular epidemiology for malaria control in high burden regions and the limitations of classic population genetic analysis in these settings, the performance of amplicon deep sequencing to enhance the understanding of malaria epidemiology in a key region for malaria elimination and control was evaluated. *P. falciparum* genetic diversity at two time points in June-July 2016 and January-March 2017 in Nchelenge District, Zambia and Kilwa and Kashobwe, the DRC is described. Genetic relatedness of parasites from northern Zambia and southern DRC were compared in order to assess whether this border region represents one contiguous transmission foci. The impact of declining parasite prevalence in Haut-Katanga Province on parasite population genetics was evaluated over the two time periods of the study. Finally, methods for detecting epidemiologically relevant genetic signals amidst high genetic diversity in a holoendemic transmission setting are discussed.

## Methods

### Sample collection and DNA extraction

At two timepoints, between June and July 2016 (dry season) and between January and March 2017 (rainy season), randomly selected households in Nchelenge District, Zambia and two villages, Kilwa and Kashobwe, in Haut-Katanga Province, the DRC were enrolled in a cross-sectional survey. Consenting individuals living within enrolled households participated in a demographic survey and had finger prick blood collected as dried blood spots (DBS) on filter paper (Whatman 903™ Protein Saver Card). DBS were stored with desiccant in sealed plastic bags and transported to the Johns Hopkins Bloomberg School of Public Health in Baltimore, Maryland, USA. DNA was extracted from the DBS using 20% Chelex and the concentration of *P. falciparum* specific DNA was quantified by qPCR targeting a fragment of the *Pfldh* gene using previously published primers [28] with SYBR green chemistry (Applied Biosystems/Thermo Fisher Scientific, Foster City, CA).

### Amplicon library preparation

*Plasmodium falciparum* positive DNA extracts were amplified at the *Pfama1* and *Pfcsp* loci. *Pfcsp* amplicon generation in 2016 was previously described [29]. *Pfama1* amplicons in 2016 were similarly generated. In brief, primers (forward primer: CCAACAAAACCTCTTATGTCACCA; reverse primer: TTAGGTTGATCCGAAGCACTCA) were fused with Illumina adapter sequences and were used to amplify a 454-basepair (bp) region of *Pfama1* [30]. *Pfama1* amplification reaction components included 9.25 μL of the extracted template DNA, 12.5 μL KAPA Hifi HotStart ReadyMix (Kapa Biosystems, Wilmington, Massachusetts), 0.25 μL of the 20 μM reverse primer, 1.0 μL of the 5 μM forward primer, and 2 μL of 25 μM magnesium chloride. Amplicon generation cycling conditions were: 95 °C for 5 min, then 32 cycles of 98 °C for 30 sec, 58 °C for 1 min, and 72 °C for 1 min, followed by 72 °C for 5 min, and 4 °C thereafter. Amplicon sizes were confirmed using TapeStation (Agilent 4200, Santa Clara) and purified using AMPure beads (Beckman Coulter, Brea, California). Unique Nextera (Illumina, San Diego, California) indexes were added to amplicons in a subsequent PCR reaction described by Illumina [30]. Indexed amplicons underwent an additional TapeStation verification and AMPure bead purification. Confirmed amplicons were quantified using PicoGreen (ThermoFisher Scientific, Waltham, Massachusetts), diluted, and combined in equimolar volumes into a single pool comprised of 96 samples for 300-bp paired-end sequencing on the Illumina MiSeq platform at the Johns Hopkins School of Medicine Sequencing and Synthesis Facility. Samples from 2017 were prepared for sequencing as described above with the following alterations: KAPA Pure Beads (Roche, Basel, Switzerland) were used for post-amplification clean-up instead of AMPure beads, Quant-iT (ThermoFisher Scientific, Waltham, Massachusetts) was used to quantity sample concentrations instead of Pico-Green, and samples were sequenced at the University of North Carolina School of Medicine High Throughput Genomic Sequencing Facility.

### Bioinformatic processing

Bioinformatic processing was done for both sequencing runs at the same time using the same parameters. Forward and reverse read pairs were stitched, sorted by primer sequence, filtered for quality (any reads with > 75% of quality scores less than 20 were excluded), and collapsed into unique parasite haplotypes within an individual using SeekDeep (January, 9, 2019) [31]. Haplotypes abundant at less than 1% within an individual were excluded. Finally, samples with less than 200 sequencing reads, or less than the 10th percentile of all reads, were excluded from the final analysis. ArcGIS, version 10.6 (ESRI, Redlands, CA) was used to map the locations of the samples used in subsequent analysis [32].

### Rarefaction

Sequencing was performed in two different facilities in 2016 and 2017. In order to ensure that potential differential read depth by sequencing facility did not bias these estimates of population diversity by year, the R package, RTK, was used to perform a rarefaction analysis [33]. Rarefaction is a process by which sequencing reads are re-sampled to a uniform, lower read depth equivalent to the lowest read coverage in the study. Diversity metrics can be computed for each re-sampling iteration to compare the raw data to the rarefied distribution and test whether read depth is responsible for higher diversity among samples with higher sequencing coverage. To test whether differential read depth biased the diversity metrics, 1000 rarefaction re-sampling replicates were performed for each amplicon, each time sampling raw sequencing reads to the read depth of the sample with the lowest read coverage in the study. For each replicate, the MOI was calculated for each individual using the rarefied sample and compared MOI estimates from the raw and rarefied data. MOI was determined by calculating number of unique *Pfcsp* or *Pfama1* haplotypes for each individual.

### Genetic diversity

Software DnaSP (version 6.11.01) was used to calculate the number of unique *Pfama1* and *Pfcsp* haplotypes in each country, nucleotide diversity (π) within each country and among all samples, and the average number of nucleotide differences between samples from within and between countries. DnaSP was used to calculate haplotype diversity (Hd), which equals 1 in a population where every haplotype is unique and 0 in a population where every haplotype is identical. Genetic diversity metrics were compared across years and sampling locations.

### Genetic distance, population structure, and differentiation by country

FASTA files containing sequences for each parasite haplotype were aligned using Geneious (version 9.1.5) (https://www.geneious.com). Geneious was used to calculate pairwise relatedness between parasite pairs as the proportion of identical single nucleotide polymorphisms (SNPs). Pairwise comparisons were then grouped into two categories depending on whether they occurred between two parasites isolated from individuals living in the same country or between two parasites within individuals residing in different countries. Mean within-country relatedness was compared between parasite pairs to the mean between-country relatedness between parasite pairs, both in an unstratified analysis as well as an analysis stratified by age. To detect signatures of population structure between parasites from Zambia and the DRC, the R package, adegenet [34] was used to conduct a discriminant analysis of principal components (DAPC) [35]. DAPC seeks to detect the linear axes which explain the most between-group variability in the data, unlike classical principal components analysis (PCA) which identifies the linear axes that explain the most variability overall [35]. DAPC was performed using country of origin as group priors. Templeton, Crandall, and Sing (TCS) haplotype networks [36] were constructed for each amplicon and visualized using tcsBU [37]. Finally, software DnaSP was used to calculate F_ST_ and test for population differentiation between *P. falciparum* isolates from Zambia and the DRC at each of the *Pfama1* and *Pfcsp* loci.

### Genetic diversity following ITN scale-up and parasite prevalence decline

Parasite population genetic diversity indicators were compared before and after the scale-up of ITNs in both the DRC and Zambia to determine whether the parasite population was genetically bottlenecked by the decline in transmission. MOI was calculated as the higher of the number of unique *Pfama1* or *Pfcsp* haplotypes for each individual and compared MOI estimates from 2016 and 2017 in Zambia and the DRC. Population bottlenecks in small populations have been associated with a loss of rare alleles [38]. In fact, such recently bottlenecked populations have been shown to exhibit a characteristic mode-shift in their allele frequency distributions, such that rare alleles cease to be the most common allele type following a bottleneck [38]. Allele frequency distributions were calculated using segregating loci to detect evidence of a mode-shift resulting in a loss of rare alleles. In addition to considering the loss of rare alleles as an indicator of a recent population bottleneck, it was determined whether there was loss of rare haplotypes between 2016 and 2017. Rare haplotypes were defined in two ways: first, rare haplotypes were considered to be those present at 2% or less in the population. Next, rare haplotypes were considered as those occurring just once in a population, hereafter referred to as “singletons.” The proportion of *Pfama1* and *Pfcsp* haplotypes that were rare in 2016 and 2017 in both countries were compared by each of these two definitions.

### Population structure from rare variants

In high transmission settings, it is challenging to identify epidemiologically relevant signals in genetic data given the high background genetic diversity. Particularly for genes like *Pfama1* and *Pfcsp* which are thought to be under balancing selection, genetically similar variants found in different populations could reflect frequency-dependent selection rather than true population panmixia. Studies using human genomes have revealed that rare variants tend to be more geographically informative [39]. In fact, analysing rare variants in the human exome has been shown to more accurately detect signatures of recent population structuring events [40]. Given that high genetic diversity among genes under balancing selective pressure could lead to the false conclusion that populations are panmictic, it was assessed whether analysing rare variants alone led to detection of population structure between countries. For both *Pfama1* and *Pfcsp*, 100 binning thresholds ranging from the minimum to the maximum frequency of haplotypes across all samples were randomly selected. For each of the 100 binning thresholds, the data was subset to include only rare haplotypes present at less than or equal to the binning threshold. Using the subset data, F_ST_ between Zambia and DRC was calculated using the R package hierfstat [41]. To verify that the inferences using rare variants were not solely driven by the smaller sample size from selecting rare haplotypes the analysis was repeated for both amplicons 1000 times, each time randomly permuting the country of origin associated with each rare haplotype.

## Results

### Amplicon generation and sequencing

**Fig. 1:**
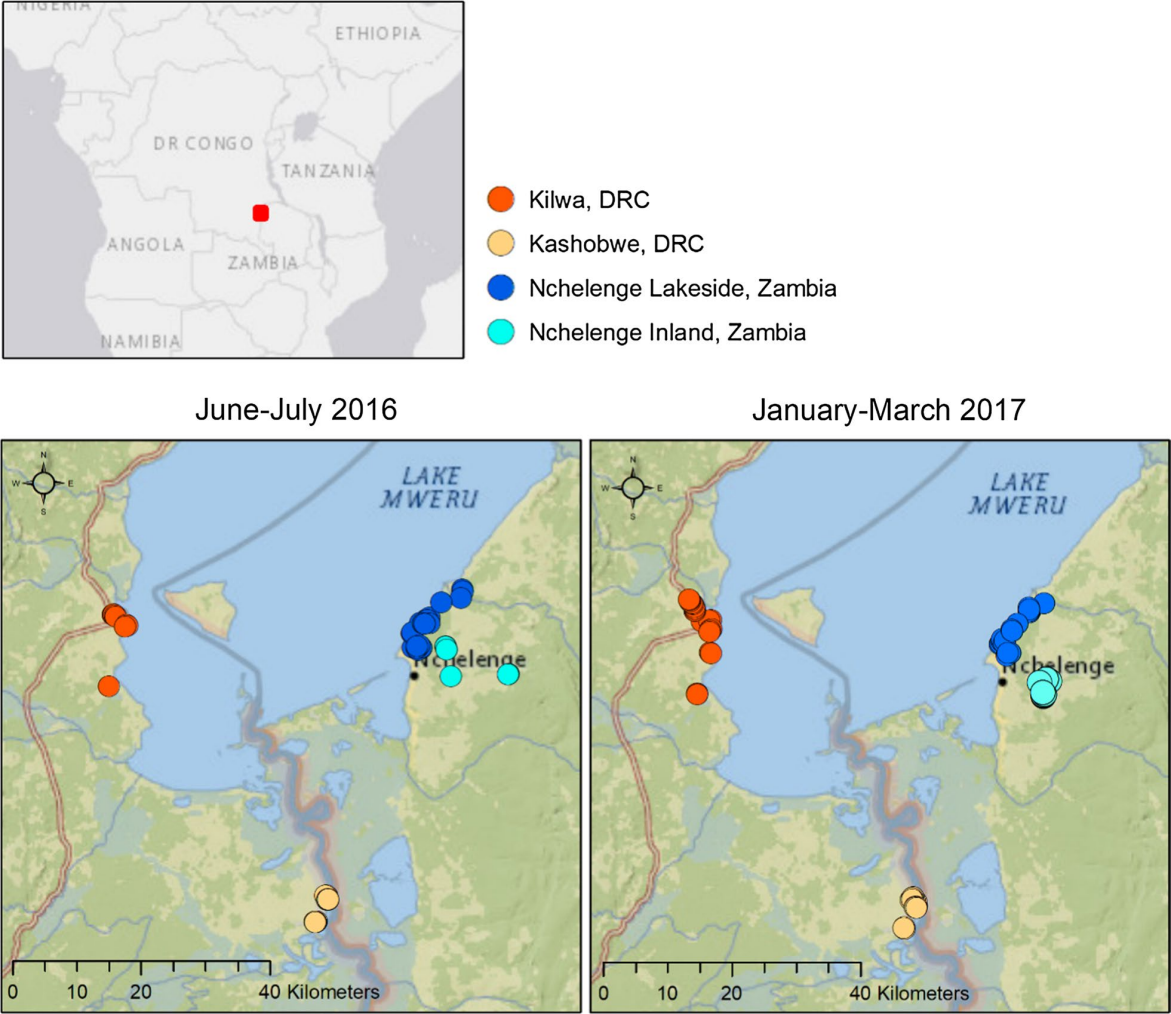
Locations of sequenced samples in 2016 (left) and 2017 (right). Samples came from two villages in Haut-Katanga Province, DRC, Kilwa (organge dots) and Kashobwe (yellow dots) and at two sites within Nchelenge District Zambia, along Lake Mweru (dark blue dots) and inland (aqua dots). Basemap Imagery Sources: National Geographic, Esri, DeLorme, HERE, UNEP-WCMC, USGS, NASA, ESA, METI, NRCAN, GEBCO, NOAA, iPC

One hundred seventy-five samples underwent amplicon generation at both the *Pfama1* and *Pfcsp* loci. Of those 175 samples, the 172 that yielded successful amplicons for *Pfama1* and *Pfcsp* loci were sequenced. Among the 172 samples sequenced, 160 (DRC 2016 = 38, DRC 2017 = 47, Zambia 2016 = 27, Zambia 2017 = 48) resulted in sequencing reads passing quality filtering for *Pfama1* and 144 (DRC 2016 = 30, DRC 2017 = 46, Zambia 2016 = 20, Zambia 2017 = 48) for *Pfcsp*. In total, 554 *P. falciparum* sequences representing 117 unique haplotypes were characterized at the *Pfama1* locus and 592 sequences representing 83 unique haplotypes at the *Pfcsp* locus (Table 1). The locations of the 2016 and 2017 sequenced samples are shown in Fig. 1.

**Table 1:** Genetic diversity by country and year

	DRC (2016 and 2017)	DRC (2016)	DRC (2017)	Zambia (2016 and 2017)	Zambia (2016)	Zambia (2017)	Overall
*Pfama1* (454 bp)							
# Sequences	301	131	170	255	78	176	554
# Segregating sites (S)	38	38	34	38	38	37	38
Nucleotide diversity (π)	0.024	0.025	0.024	0.024	0.024	0.024	0.024
Nucleotide diversity (π): non-synonymous sites	0.029	0.030	0.029	0.029	0.030	0.030	0.029
# Unique haplotypes	82	53	62	87	46	72	117
Haplotype diversity (Hd)	0.975	0.976	0.974	0.978	0.978	0.978	0.977
Average # nucleotide differences (K)	10.995	11.135	10.916	10.951	10.755	11.044	10.977
*Pfcsp* (253 bp)							
# Sequences	303	106	197	288	66	222	592
# Segregating sites (S)	33	29	32	30	25	30	35
Nucleotide diversity (π)	0.024	0.024	0.024	0.025	0.025	0.026	0.025
Nucleotide diversity (π): non-synonymous sites	0.030	0.030	0.030	0.032	0.031	0.032	0.031
# Unique haplotypes	62	43	55	62	31	56	83
Haplotype diversity (Hd)	0.966	0.967	0.967	0.966	0.961	0.968	0.966
Average # nucleotide differences (K)	6.052	6.101	6.033	6.418	6.231	6.483	6.214

DnaSP (version 6.10.01) was used to calculate the number of segregating sites (S), nucleotide diversity (π) among all loci, nucleotide diversity (π) among non-synonymous sites, the number of unique DNA haplotypes, haplotype diversity (Hd), and the average number of nucleotide differences between *P. falciparum* haplotype for each of the populations

### Rarefaction analysis

Differential read coverage was observed between the *Pfcsp* amplicons from 2016 and 2017 sequencing runs. In 2016, amplicons were supported by an average of 52,600 reads for *Pfama1* and 639 for *Pfcsp.* In 2017, samples were supported by an average of 41,813 reads for *Pfama1* and 47,134 reads for *Pfcsp.* To test whether the lower read coverage in 2016 for *Pfcsp* could bias the estimates of genetic diversity, rarefaction analysis was performed to a read depth of 200 (the lowest read depth among the samples). Additional file 1: Figure S1 displays collector’s curves for each amplicon starting from either the raw data or the rarefied data. Collector’s curves show the increase in the number of unique haplotypes observed as more randomly selected samples are considered. For both amplicons, the collector’s curves are identical starting from either the raw data or the rarefied data and performing 1000 replicates of the collector’s curve analysis. This suggests that the raw data contains no more diversity than that which is captured in the rarefied subsample. Further, MOI estimates for both amplicons are nearly identical regardless of whether the estimate was derived from the raw or rarefied data (*Pfama1* regression R^2^ = 0.99; *Pfcsp* regression R^2^ = 0.99) (Additional file 1: Figure S2), suggesting that the raw and rarefied datasets are equivalent in the diversity they explain. The total genetic diversity present among all sequencing reads was captured even when 200 reads per sample were used. Upon this demonstration that the lower read depth observed in 2016 did not bias the diversity estimates, remaining analyses were conducted using the unrarefied data.

### Genetic distance, population structure, and differentiation by country

Genetic diversity was high in both Zambia and the DRC across both time points in 2016 and 2017. Among the samples, high Hd (Table 1) was observed across both countries and timepoints, highlighting the high degree of genetic diversity in these high transmission settings. Diversity, as measured by Hd and nucleotide diversity, remained high across both time points, with no significant differences by country or time (Table 1).

**Fig. 2:**
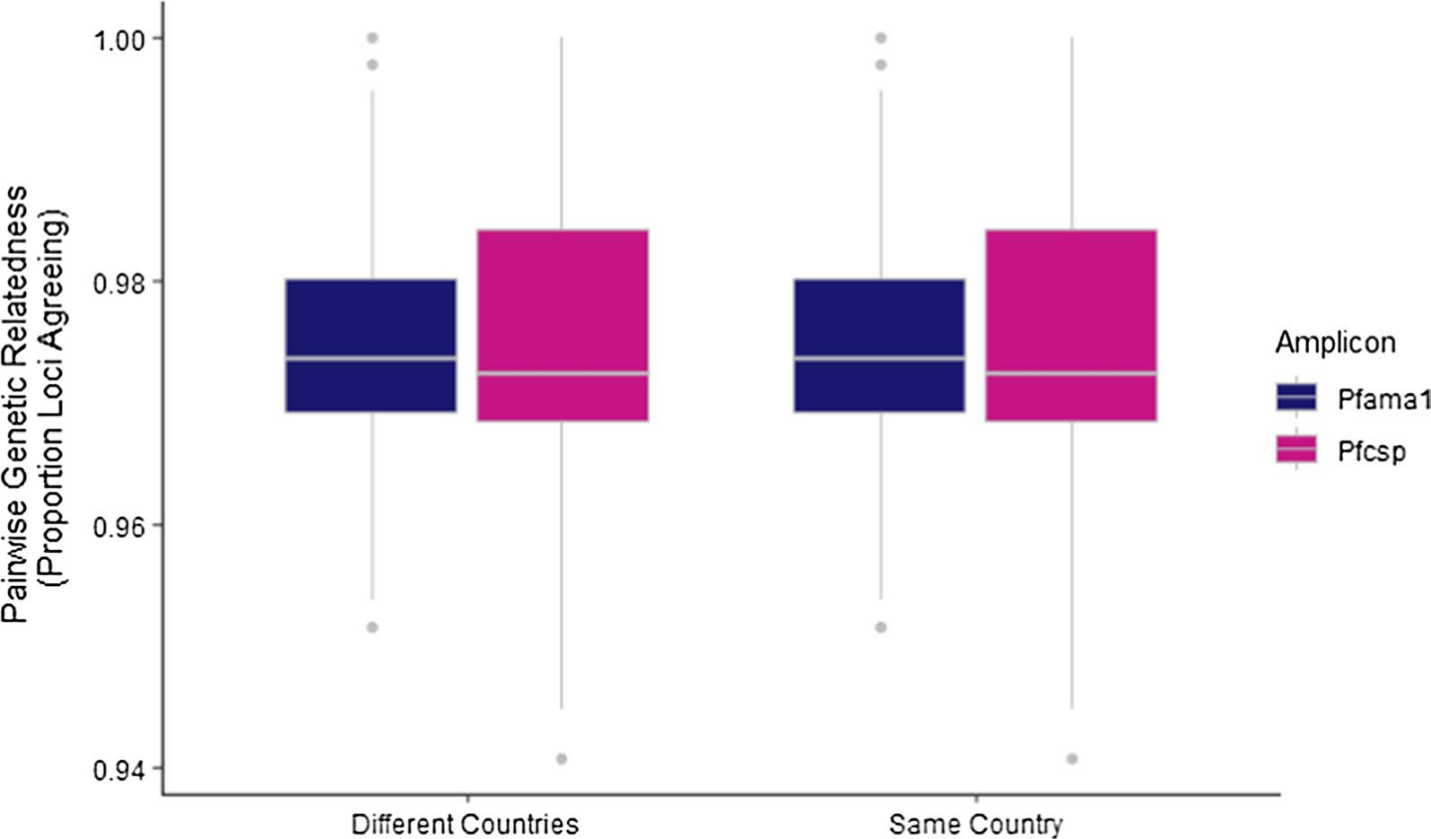
Pairwise genetic relatedness (the proportion of loci that match) is plotted for all pairs of parasites from different countries (left) or from the same country (right). *Pfama1* comparisons are shown in blue and *Pfcsp* comparisons are shown in pink

**Fig. 3:**
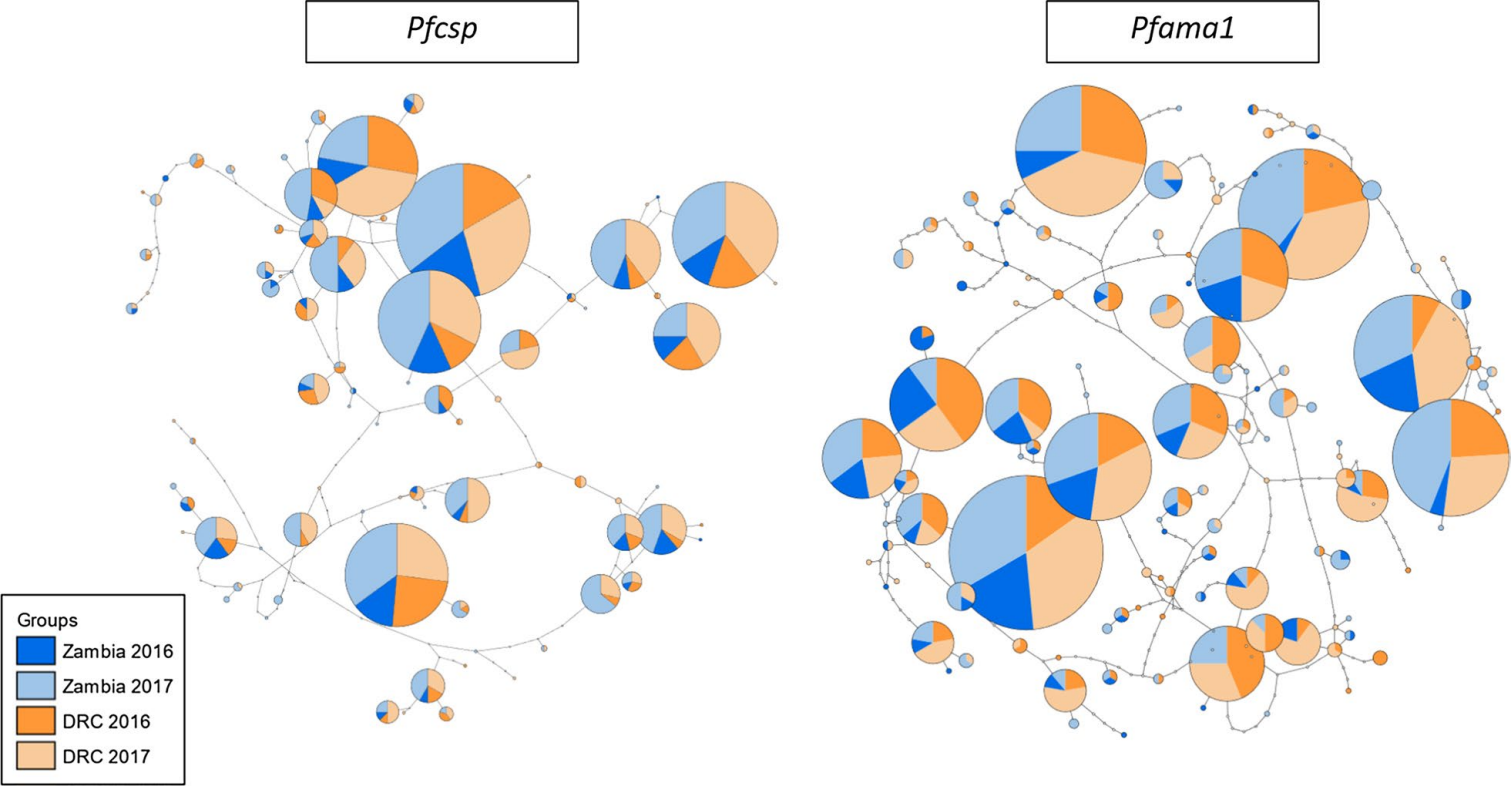
TCS haplotype networks for *Pfcsp* (left) and *Pfama1* (right). Each circle represents a unique haplotype; circles are scaled according to the frequency each haplotype was observed and colored by the proportion of sequences per haplotype originating from Zambia (blue) or the DRC (orange). Darker shades indicate that samples were collected in 2016, and lighter shades indicate that samples were collected in 2017. The number of mutations that differ between haplotypes is indicated by the number of notches in the lines connecting circles

Further, after calculating pairwise genetic relatedness between all pairs of parasites, there was no significant difference in relatedness comparing parasites from within the same country to parasites from different countries for either *Pfama1* or *Pfcsp* (Fig. 2). Age stratified analysis was similar to the unstratified analysis (Additional file 1: Figure S3). In genetically differentiated populations, within-country relatedness is expected to be higher than between-country relatedness, which was not demonstrated here. Similarly, the most common haplotypes in the study were shared at similar frequencies in both Zambia and the DRC (Fig. 3). DAPC analysis failed to identify a linear axis which could reliably discriminate isolates from Zambia and the DRC at either *Pfama1* or *Pfcsp* (Additional file 1: Figure S6A, C), suggesting that these populations are not genetically distinct. Further, F_ST_ between the countries was found to be 0.00205 for *Pfama1* and 0.00023 for *Pfcsp,* suggesting no evidence of population structure between countries. Finally, DnaSP detected no statistically significant population differentiation between parasites from Zambia and the DRC at either the *Pfama1* (p = 0.10) or *Pfcsp* (p = 0.15) loci. Together, these observations are consistent with the hypothesis that parasites from Zambia and the DRC represent a single, highly genetically diverse population.

### Population bottleneck analysis

**Fig. 4:**
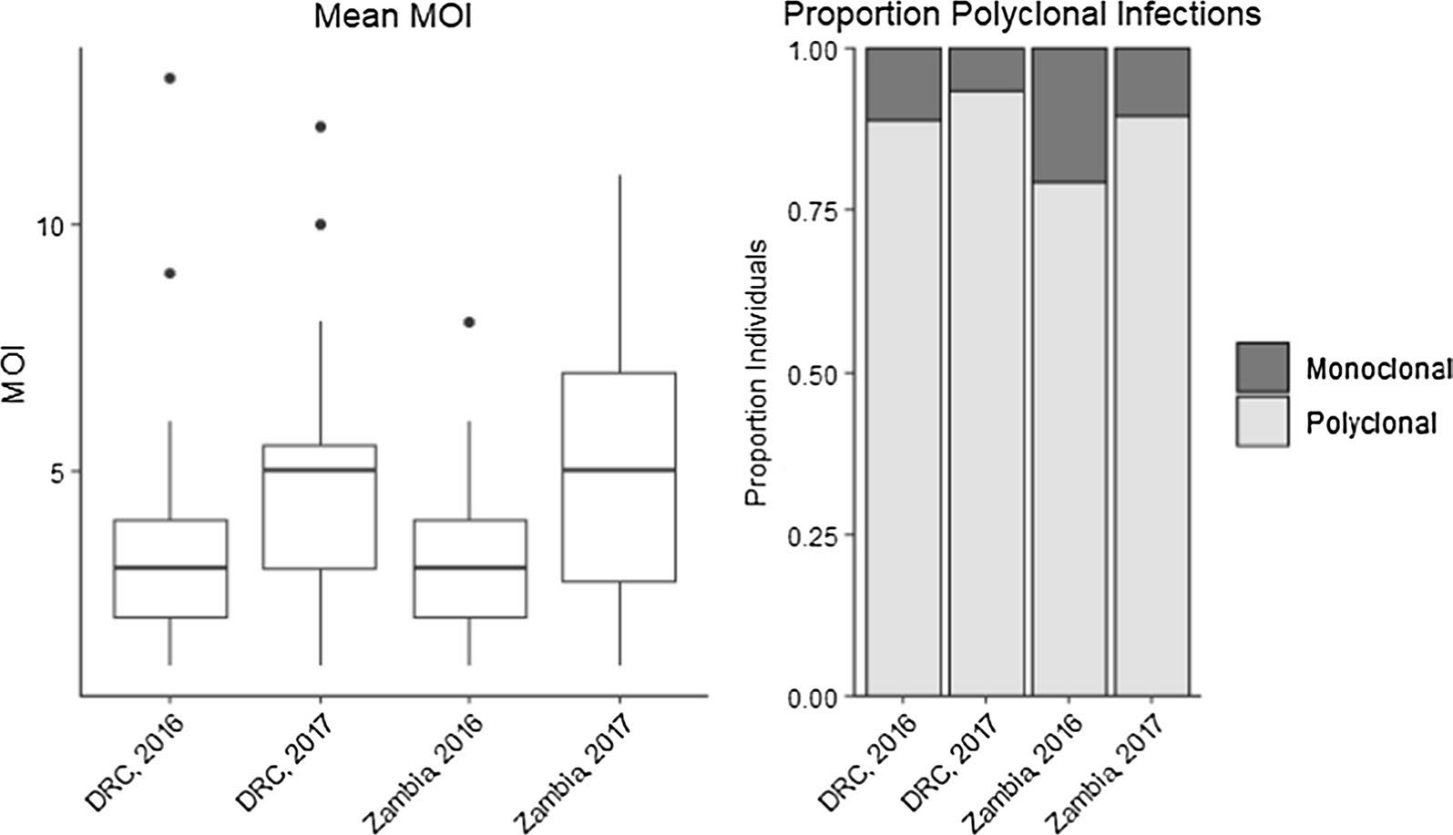
MOI was determined to be the higher of the total number of unique haplotype present within an individual at either the *Pfcsp* or *Pfama1* loci. Individuals were considered monoclonal if their MOI was estimated to be 1 and polyclonal if their MOI was > 1

**Fig. 5:**
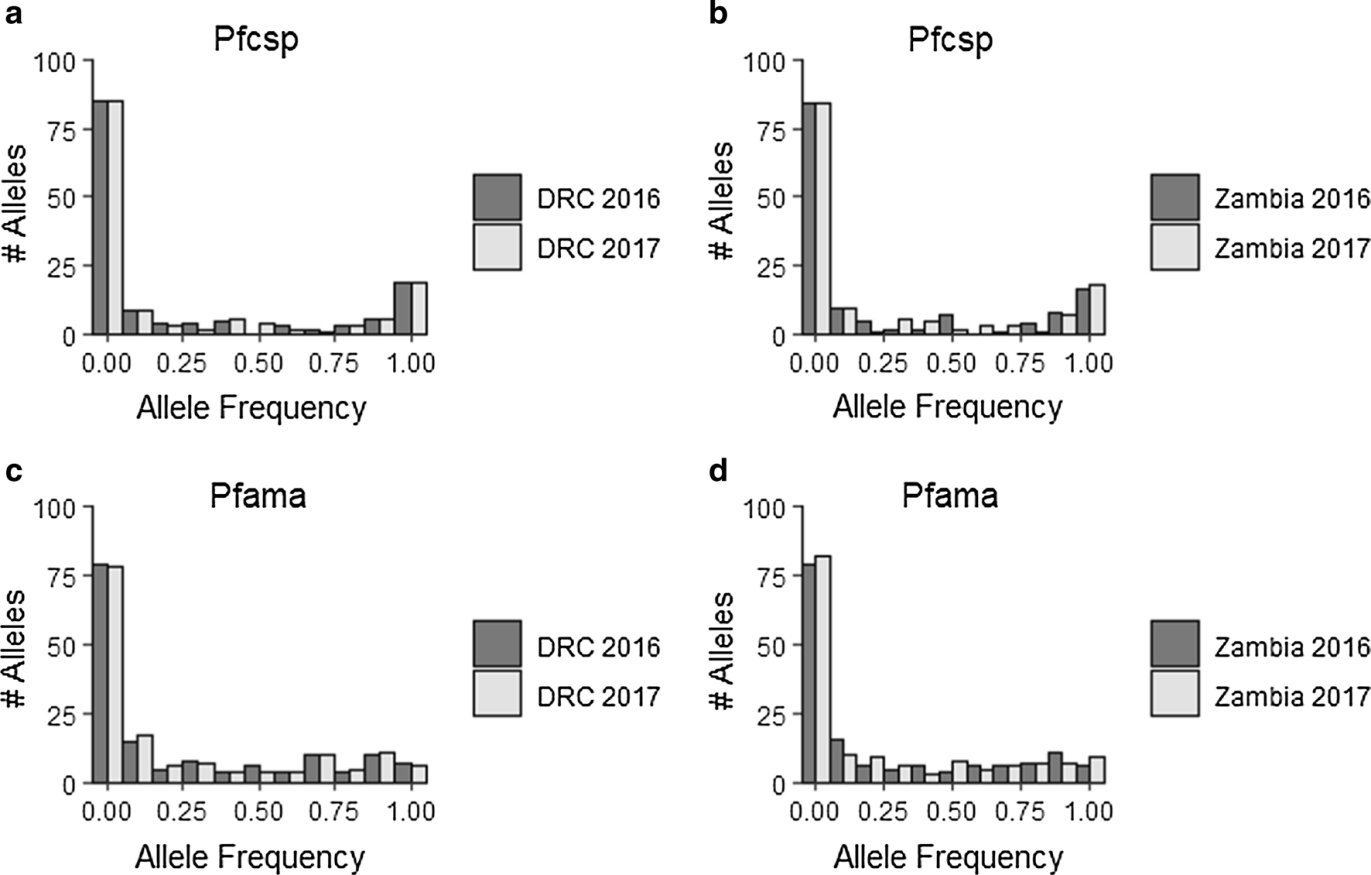
The allele frequency distribution is plotted for each population (**a**, **c** DRC; **b**, **d** Zambia; light grey bars: 2016 samples; dark grey bars: 2017 samples). Frequencies were calculated considering loci which were found to be segregating sites in the total dataset (*Pfcsp*: n = 35; *Pfama1*: n = 38)

Multiple studies have reported declines in MOI that accompany decreases in transmission (12, 14, 17), but consistent with the other findings of this study, MOI did not decrease among individuals from the DRC in 2017 following the ITN distribution (DRC 2016: MOI = 3.78; DRC 2017: MOI = 4.64) (Fig. 4). Declines in parasite prevalence may result in a population bottleneck when comparing isolates from before (n = 131 *Pfama1* isolates and n = 106 *Pfcsp* isolates in the DRC 2016) and after (n = 170 *Pfama1* sequences and n = 197 *Pfcsp* sequences in the DRC 2017) the ITN distribution. There was no evidence of an allele frequency mode-shift indicative of a population bottleneck comparing the DRC isolates from 2016 and 2017. Similarly, a mode-shift was not detected from the haplotype frequency distributions either (Additional file 1: Figure S4). In fact, the proportion of haplotypes classified as rare by multiple thresholds was similar across countries and timepoints (Additional file 1: Figure S5) (Fig. 5).

### Population structure from rare variants

**Fig. 6:**
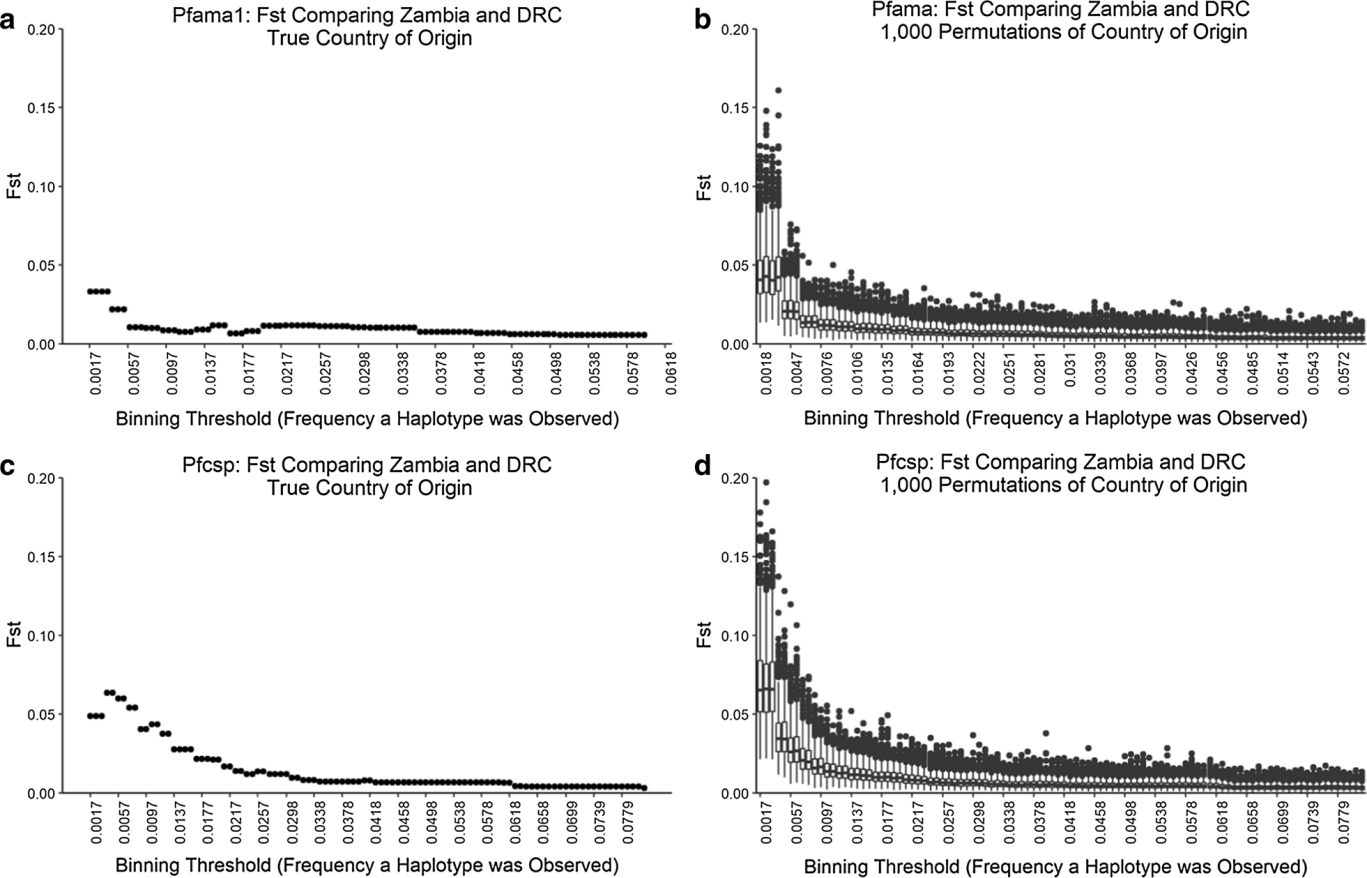
**a**, **b** For each of 100 randomly selected binning threshold (X-axis) ranging from the minimum to the maximum haplotype frequencies for each amplicon (**a** *Pfama1*, **b** *Pfcsp*), we classified parasites as rare or not, subset the data to include only rare parasites, and calculated F_ST_ (Y-axis) between Zambia and the DRC using only the subset data. **c**, **d** To test whether the reduced sample size was driving patterns in F_ST_ we randomly permuted the country of origin for each sample 1000 times. For each permutation, we subset the data to include only rare samples based on each binning threshold, and calculated F_ST_ comparing Zambia and the DRC. Boxplots show the range of F_ST_ estimates across the 1000 permutation replicates for *Pfama1* (**c**) and *Pfcsp* (**d**)

Using only rare variants, a possible signature was observed of low to moderate population structuring (F_ST_ = 0.06) between *Pfcsp* sequences from Zambia and the DRC (Fig. 6c). The F_ST_ estimates for the binning thresholds that included the rarest *Pfama1* samples were low (F_ST_ = 0.025) and not indicative of population differentiation (Fig. 6a). However, although F_ST_ values were possibly indicative of genetic differentiation between Zambia and the DRC using rare *Pfcsp* isolates, the possibility that this observation was due to the small sample size remaining after subsetting rare *Pfcsp* haplotypes cannot be excluded. In fact, the F_ST_ value calculated from rare *Pfcsp* variants was not significantly different from the distribution of F_ST_ values obtained from randomly permuting the country of origin among rare haplotypes 1000 times and estimating F_ST_ (Fig. 6b, d). Further, DAPC using only rare variants resulted in improved population separation between Zambia and the DRC, particularly for *Pfcsp* (Additional file 1: Figure S6B, D). This was true when rare haplotypes were defined as singletons (Additional file 1: Figure S6) or as those present at 2% or less in the population (Additional file 1: Figure S7).

## Discussion

The utility of parasite genotyping to enhance malaria epidemiology has been well-demonstrated in low to moderate transmission settings. Although parasite genotyping has been useful in assessing the prevalence of drug resistance mutations in high transmission settings [42], it remains to be proven as a tool for evaluating control interventions or enhancing the understanding of transmission epidemiology in this context. In a holoendemic transmission region along the international border between Luapula Province, Zambia and Haut-Katanga Province, the DRC, high genetic *Pfama1* and *Pfcsp* diversity was observed across two different seasons, indicating that these loci are diverse and unstructured in high transmission settings. Given the benefit of using amplicon deep sequencing in such regions with high polyclonality, this observation highlights the limitations of utilizing parasite population genetic analysis to understand transmission epidemiology in high-burden areas. Although multiple studies in moderate to low transmission settings reported decreases in genetic diversity and MOI following decreased transmission [11–19], no signatures of a parasite population bottleneck were detected in this study despite an ITN distribution campaign between sampling timepoints that reduced the parasite prevalence by microscopy in Kilwa and Kashobwe, the DRC using amplicon deep sequencing of two highly diverse antigens. This reflects the fact that transmission remains high in this region even after the observed decrease in parasite prevalence, and the parasite population remains sufficiently large and, therefore, shielded from a genetic bottleneck. Further, the choice of non-neutral genetic loci, which enabled haplotypic characterization of polyclonal infections, may have hindered the ability of this study to detect changes in population genetic diversity, underscoring the current limitations of implementing molecular epidemiologic approaches in high-burden transmission settings. It is also possible that genetic signatures of a population bottleneck may take longer than 6 months to become apparent, and were missed in this study. A much larger decline in transmission is likely required to bottleneck the parasite population. Further, although parasite genotyping has been touted as a tool for monitoring changes in transmission intensity and evaluating control interventions [7, 8], these utilities may be limited to moderate and low transmission settings, and may be less useful in high transmission regions, where high polyclonality necessitates analysis of non-neutral markers.

No evidence of population structure was detected comparing *Pfama1* and *Pfcsp* isolates between Zambia and the DRC. While clear signatures of population differentiation are easily interpretable, it is more challenging to attribute cause for observations with no discernable population structure. A lack of population structure could either reflect the true underlying biology of an admixed population or may be an artifact of the use of genetic markers that are under selection and alone not ideal for assessing population structure on a small spatial scale in high transmission zones [43, 44]. Although the same *Pfcsp* amplicon that was examined in this study revealed parasite population genetic structure on a continental scale [20], additional research is merited to assess the utility of these *Pfama1* and *Pfcsp* amplicons to detect population structure on smaller geographic scales. While these data suggest *P. falciparum* parasites from Nchelenge, Zambia and Kilwa and Kashobwe, the DRC, exist as a single panmictic population, increasing either the number of neutral SNPs characterized or the number of isolates sequenced could reveal finer scale population structuring.

It is typical to analyse neutral, unlinked SNPs in population genomic analysis. This study characterizes two highly variable *P. falciparum* genes, *Pfama1* and *Pfcsp* which are known to be under balancing selective pressure [45]. If balancing selection were to occur independently at geographically separated sites, then the isolation by distance signal could be attenuated, which would lead to the inability to correctly identify population structure when it truly exists. Further, since *Pfama1* and *Pfcsp* are under balancing selection, changes in their diversity do not necessarily reflect changes in transmission. Finally, the SNPs within each of these two amplicons are in linkage disequilibrium in the *P. falciparum* genome. It is possible that the use of non-neutral, linked loci biased these analyses such that true population differentiation was not detected between Nchelenge District, Zambia and Haut-Katanga Province, the DRC or failed to detect genetic signatures of a population bottleneck. However, in regions where most infections are comprised of multiple, genetically distinct parasite clones, amplicon deep sequencing is perhaps the most cost-effective method capable of preserving parasite haplotypes, bypassing the need to invoke potentially biased haplotype reconstruction methods or discarding polyclonal infections prior to analysis. In *P. falciparum* genetics, it has been common practice to exclusively analyse monoclonal infections [11, 46, 47] or disregard loci where two or more alleles are present in polyclonal infections [48, 49]. While such practices may be appropriate in some settings where MOI is low, they are not an option in high burden regions like Luapula and Haut-Katanga Provinces, where restricting an analysis to monoclonal infections would require discarding close to 80% of the data (Pringle, unpublished). As methods for handling polyclonal genetic data continue to improve, it may eventually be possible to select unlinked, neutral loci for additional analyses to assess parasite population structure in border regions and detect signatures of population bottlenecks in moderate to high transmission settings.

Despite the use of non-neutral, linked SNPs, the data suggesting a contiguous *P. falciparum* population are consistent with whole genome sequencing analyses from *Anopheles funestus* mosquitoes that did not detect population structure of vectors between Nchelenge, Zambia and Haut-Katanga Province, the DRC ([50], Lee, unpublished). These data supporting a single and large primary vector population suggest a possible mechanism that might drive the regular genetic crossing and lack of population differentiation among *P. falciparum* isolates from across the country border. The observation of a contiguous parasite population across the border between Zambia and DRC suggest that collaborative malaria control efforts targeting both regions together may enhance intervention success. Border regions of a country frequently experience higher malaria transmission than non-border regions and often harbor the final transmission foci prior to elimination [51]. The observation that Luapula Province, Zambia and Haut-Katanga Province, the DRC together comprise a contiguous high transmission foci along an international border highlights the importance of expanding existing regional partnerships [52] like Elimination 8 (E8) in southern Africa that can facilitate the coordination of elimination efforts across multiple nations. A study which looked at how frequently the Global Fund funded malaria projects aiming to establish multi-national control efforts [53] found that these proposals are rarely funded, and that there is little guidance for what makes these projects successful. Developing new strategies to guide, fund, and support regional initiatives that encourage international cooperation towards malaria elimination may enhance current and future efforts. As efforts to eliminate malaria across the globe continue to expand, addressing the unique challenge of controlling border malaria is essential.

## Conclusions

Achieving Zambia’s malaria elimination target date of 2021 will require substantially reducing the burden of malaria in the holoendemic transmission region in Luapula Province. Although parasite genotyping may be valuable in addressing specific questions, like the prevalence of drug resistance mutations, it is challenging to use parasite genotyping to draw inferences regarding transmission epidemiology in high burden regions characterized by high genetic diversity using current tools. This study explored whether analysing rare haplotypes enhanced the ability to elucidate transmission patterns in a holoendemic setting. While restricting the analysis to rare variants did lead to the detection of a possible genetic signature of population structure, it is unclear whether this signal is real, or merely an artifact from the reduced sample size. Incorporating rare variant analytical approaches in *P. falciparum* population genetic analysis may be beneficial but should be interpreted with caution when sample sizes are significantly reduced. Continued decreases in the cost of whole genome sequencing, improved computational methods for phasing sequencing reads from polyclonal data, and genetic distance metrics that account for polyclonality and high background diversity may lead to an enhanced value of parasite genotyping in high burden regions.

## Supplementary information

**Additional file 1: Fig. S1.** Top: *Pfama1*; bottom: *Pfcsp*. Distributions across 1000 replicates of collector’s curve analysis show the number of unique haplotypes (Y-axis) found among a randomly selected group of samples of increasing size (X-axis). Curves on the left were generated using the raw (unrarefied) dataset, while curves on the right were generated from the dataset rarefied to a depth of 200 reads per sample. **Fig. S2.** For each amplicon (top: *Pfama1*, bottom: *Pfcsp*), we performed 1000 re-sampling replicates of rarefaction. For each replicate we estimated MOI in each individual using the rarefied data. The distribution of MOI estimates across all rarefaction re-sampling replicates are plttoed along the Y-axis. The X-axis shows the MOI estimate from the raw data. The red dashed line is the Y = X line, or what would be expected if there was no difference between the estimate using rarefied data and the true estimate. **Fig. S3.** Pairwise genetic relatedness (the proportion of loci which are identical between two parasites) is plotted for all pairs of parasites from different countries or from the same country for each amplicon, *Pfama1* (left) and *Pfcsp* (right). Comparisons between two parasites from individuals both under 5 years old are shown in pink. Comparisons between two parasites from individuals both 5 years or older are shown in blue. Comparisons between parasites from an individual 5 years or older and an individual under 5 years are shown in yellow. **Fig. S4.** Haplotype frequency distributions are plotted for the haplotypes present in each population (left: DRC right: Zambia; light grey bars: 2016 samples; dark grey bars: 2017 samples). **Fig. S5**. Left: plots the proportion of haplotypes within the four country-year “populations” considered to be rare. Here rare haplotypes are those represented at 2% or less in the “population.” Right: plots the proportion of haplotypes within the four country-year “populations” that were observed only once (singletons). **Fig. S6.** Dicriminatory analysis of principal components (DAPC) was performed using R package, adgenet. DAPC performs linear discriminat analysis on principal components in order to maximize separation of *a priori* groups. A, B: *Pfama1*; C, D: *Pfcsp*. A, C: DAPC performed using all sequences regardless of population frequency shows no linear function that can classify the principal components of the parasite seqeunces reliably by country. B, D: DAPC using only rare haplotypes (singletons) results in more refined population discrimination for *Pfcsp*. **Fig. S7.** Dicriminatory analysis of principal components (DAPC) was performed using R package, adgenet. DAPC performs linear discriminat analysis on principal components in order to maximize separation of *a priori* groups. A, B: *Pfama1*; C, D: *Pfcsp*. A, C: DAPC performed using all sequences regardless of population frequency shows no linear function that can classify the principal components of the parasite seqeunces reliably by country. B, D: DAPC using only rare haplotypes (2% or less frequency) results in more refined population discrimination for *Pfcsp*.

## Data Availability

*Pfcsp* sequences from 2016 were previously deposited to GenBank (accession numbers: MG715504-MG715555) (29). *Pfama1* sequences from 2016 and 2017 as well as *Pfcsp* sequences from 2017 also were deposited to GenBank (accession numbers MN044107- MN044259).

## Abbreviations

DRC: Democratic Republic of the Congo
IRS: indoor residual spray
ITN: insecticide-treated bed net
ACTs: artemisinin-combination therapies
HBHI: high burden to high impact
WHO: World Health Organization
RDT: rapid diagnostic test
DHS: Demographic and Health Survey
DBS: dried blood spots
*Pfama1*: *Plasmodium falciparum apical membrane antigen 1*
*Pfcsp*: *Plasmodium falciparum circumsporozoite protein*
qPCR: quantitative polymerase chain reaction
SNP: single nucleotide polymorphism
DAPC: discriminatory analysis of principal components
TCS: Templeton, Crandall, and Sing
MOI: multiplicity of infection
