# Supplementary material for: High Plasmodium falciparum genetic diversity and temporal stability despite control efforts in high transmission settings along the international border between Zambia and the Democratic Republic of the Congo
Source: Malar J. 2019 Dec 4;18:400. doi: 10.1186/s12936-019-3023-4 (PMC6894251; doi:10.1186/s12936-019-3023-4)
Supplement: Supplementary file 1 — Additional file 1: Fig. S1. Top: Pfama1; bottom: Pfcsp. Distributions across 1000 replicates of collector’s curve analysis show the number of unique haplotypes (Y-axis) found among a randomly selected group of samples of increasing size (X-axis). Curves on the left were generated using the raw (unrarefied) dataset, while curves on the right were generated from the dataset rarefied to a depth of 200 reads per sample. Fig. S2. For each amplicon (top: Pfama1, bottom: Pfcsp), we performed 1000 re-sampling replicates of rarefaction. For each replicate we estimated MOI in each individual using the rarefied data. The distribution of MOI estimates across all rarefaction re-sampling replicates are plttoed along the Y-axis. The X-axis shows the MOI estimate from the raw data. The red dashed line is the Y = X line, or what would be expected if there was no difference between the estimate using rarefied data and the true estimate. Fig. S3. Pairwise genetic relatedness (the proportion of loci which are identical between two parasites) is plotted for all pairs of parasites from different countries or from the same country for each amplicon, Pfama1 (left) and Pfcsp (right). Comparisons between two parasites from individuals both under 5 years old are shown in pink. Comparisons between two parasites from individuals both 5 years or older are shown in blue. Comparisons between parasites from an individual 5 years or older and an individual under 5 years are shown in yellow. Fig. S4. Haplotype frequency distributions are plotted for the haplotypes present in each population (left: DRC right: Zambia; light grey bars: 2016 samples; dark grey bars: 2017 samples). Fig. S5. Left: plots the proportion of haplotypes within the four country-year “populations” considered to be rare. Here rare haplotypes are those represented at 2% or less in the “population.” Right: plots the proportion of haplotypes within the four country-year “populations” that were observed only once (singletons). Fig. S6 [file 12936_2019_3023_MOESM1_ESM.docx]

**Figure S1**

**
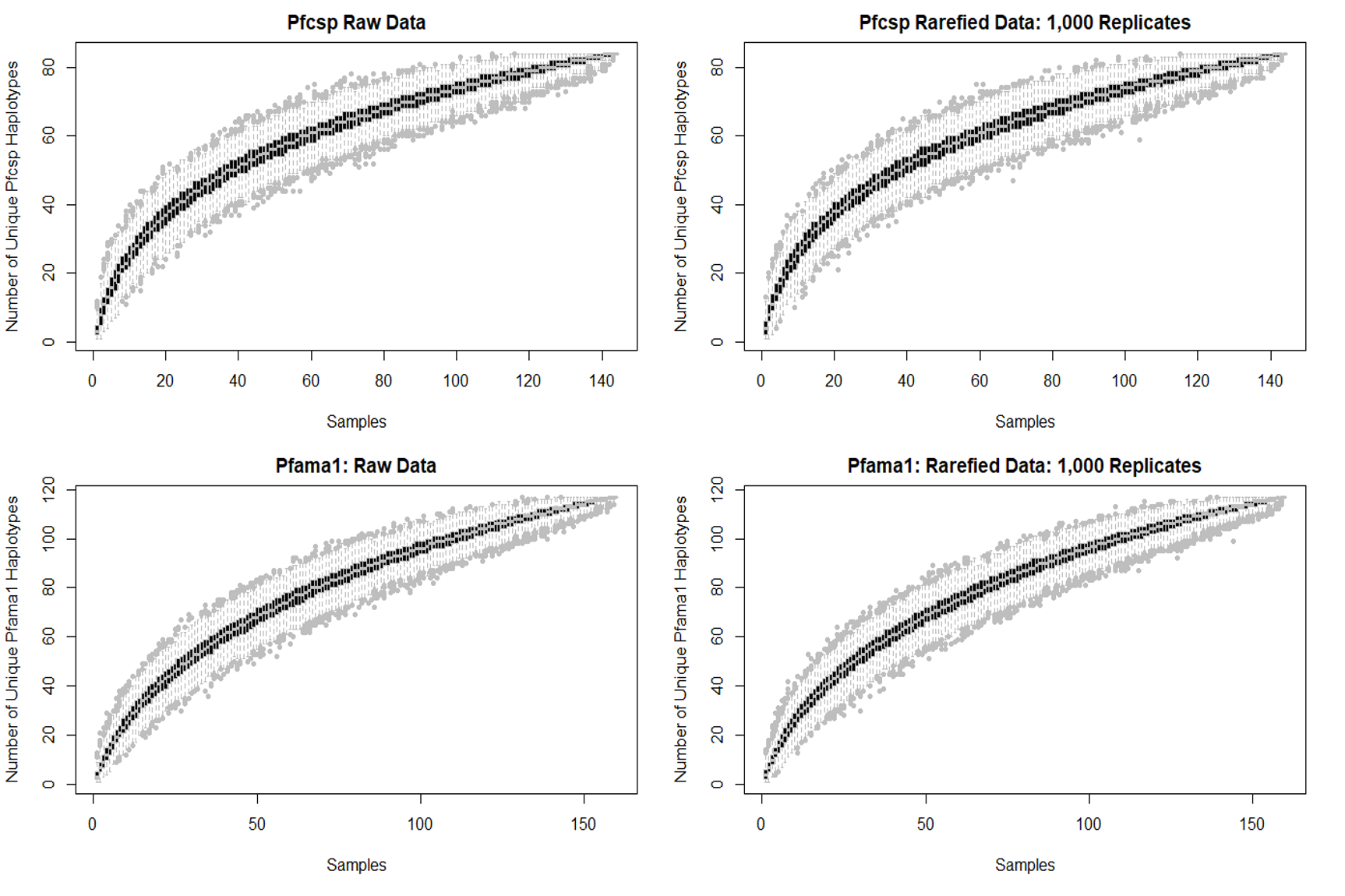
**

**Figure S1:** Top: *Pfama1*; bottom: *Pfcsp*. Distributions across 1,000 replicates of collector’s curve analysis show the number of unique haplotypes (Y-axis) found among a randomly selected group of samples of increasing size (X-axis). Curves on the left were generated using the raw (unrarefied) dataset, while curves on the right were generated from the dataset rarefied to a depth of 200 reads per sample.

**Figure S2**


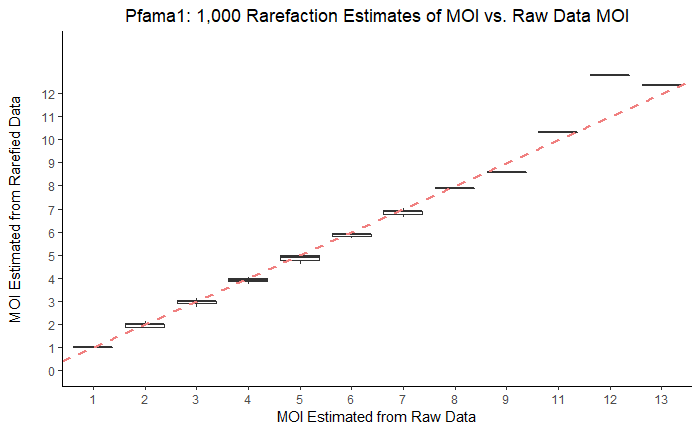

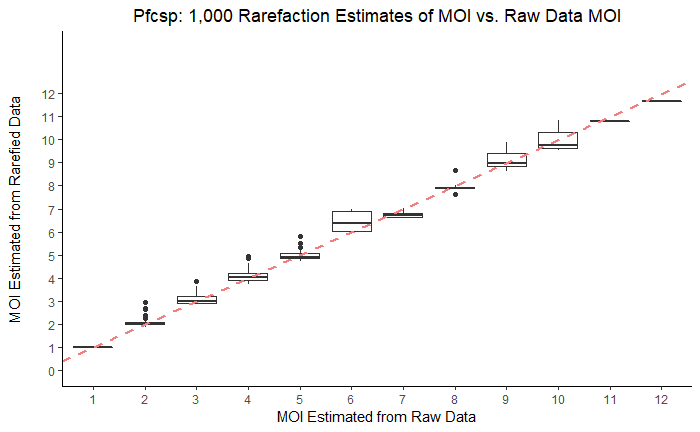


**Figure S2:** For each amplicon (top: *Pfama1*, bottom: *Pfcsp*), we performed 1,000 re-sampling replicates of rarefaction. For each replicate we estimated MOI in each individual using the rarefied data. The distribution of MOI estimates across all rarefaction re-sampling replicates are plttoed along the Y-axis. The X-axis shows the MOI estimate from the raw data. The red dashed line is the Y=X line, or what would be expected if there was no difference between the estimate using rarefied data and the true estimate.

**Figure S3:**

**
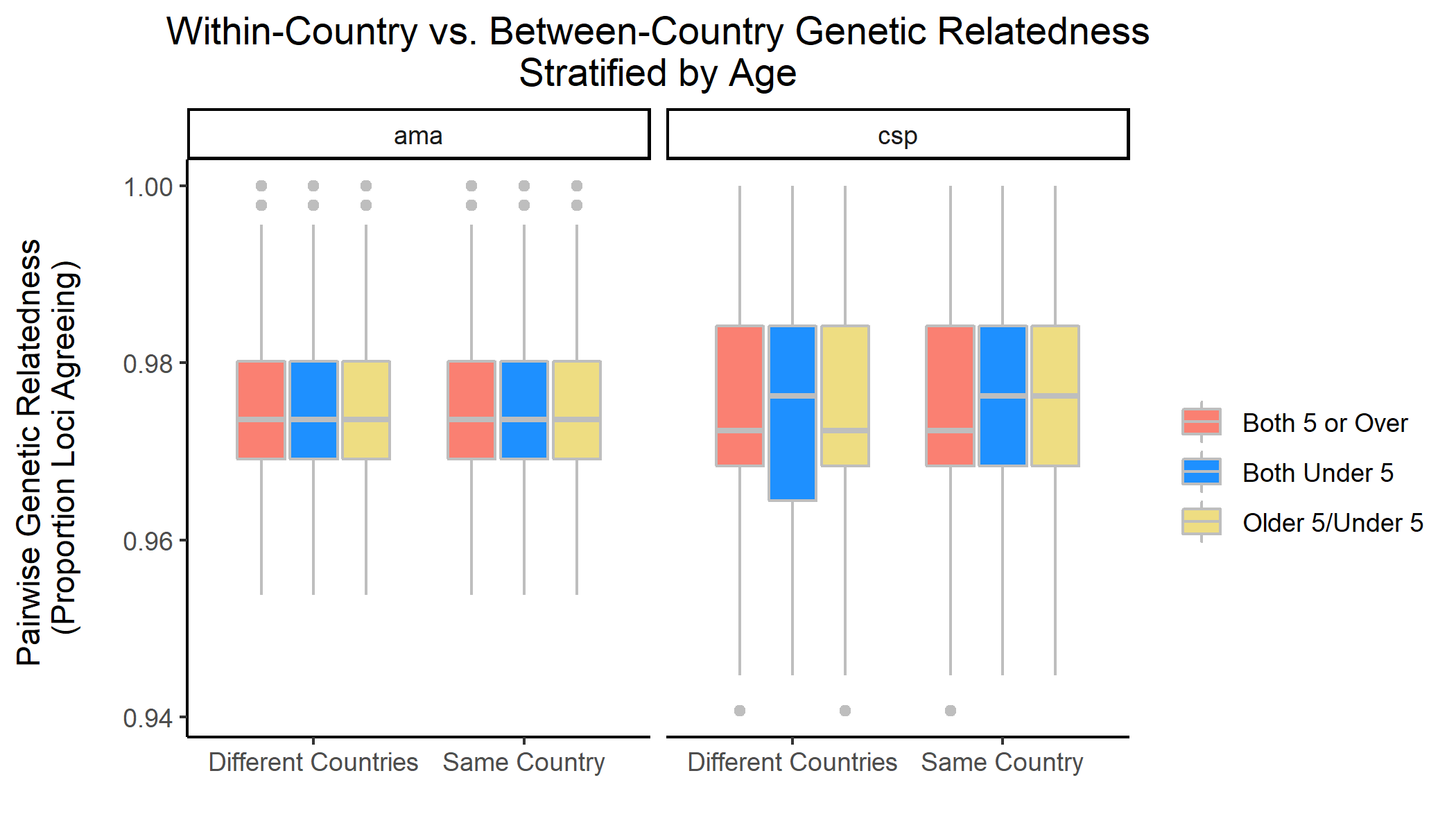
**

**Figure S3:** Pairwise genetic relatedness (the proportion of loci which are identical between two parasites) is plotted for all pairs of parasites from different countries or from the same country for each amplicon, *Pfama1* (left) and *Pfcsp* (right). Comparisons between two parasites from individuals both under five years old are shown in pink. Comparisons between two parasites from individuals both five years or older are shown in blue. Comparisons between parasites from an individual five years or older and an individual under five years are shown in yellow.

**Figure S4**


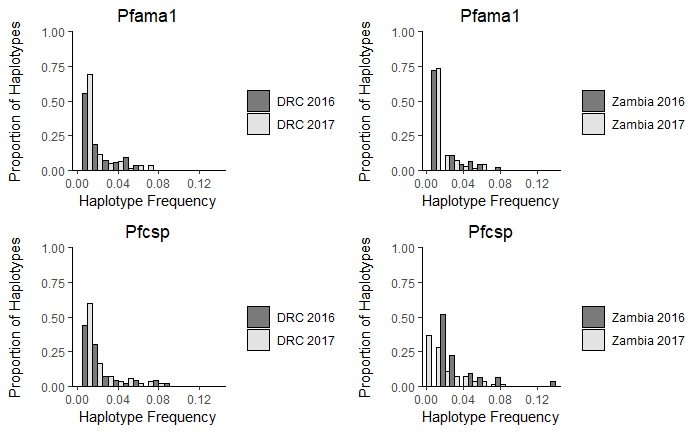


**Figure S4:** Haplotype frequency distributions are plotted for the haplotypes present in each population (left: DRC right: Zambia; light grey bars: 2016 samples; dark grey bars: 2017 samples).

**Figure S5**


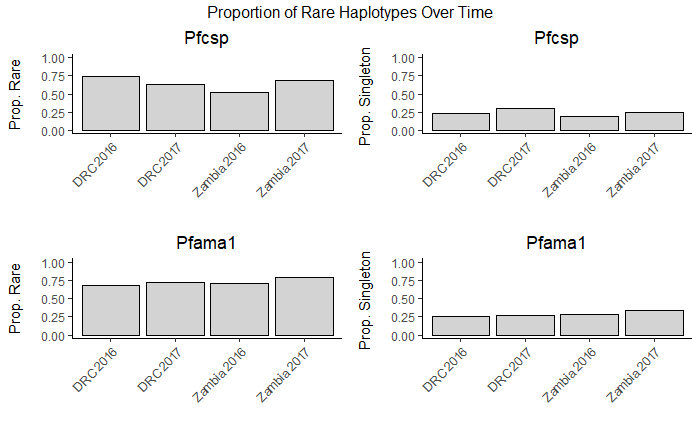


**Figure S5**: Left: plots the proportion of haplotypes within the four country-year “populations” considered to be rare. Here rare haplotypes are those represented at 2% or less in the “population.” Right: plots the proportion of haplotypes within the four country-year “populations” that were observed only once (singletons).

**Figure S6:**

**
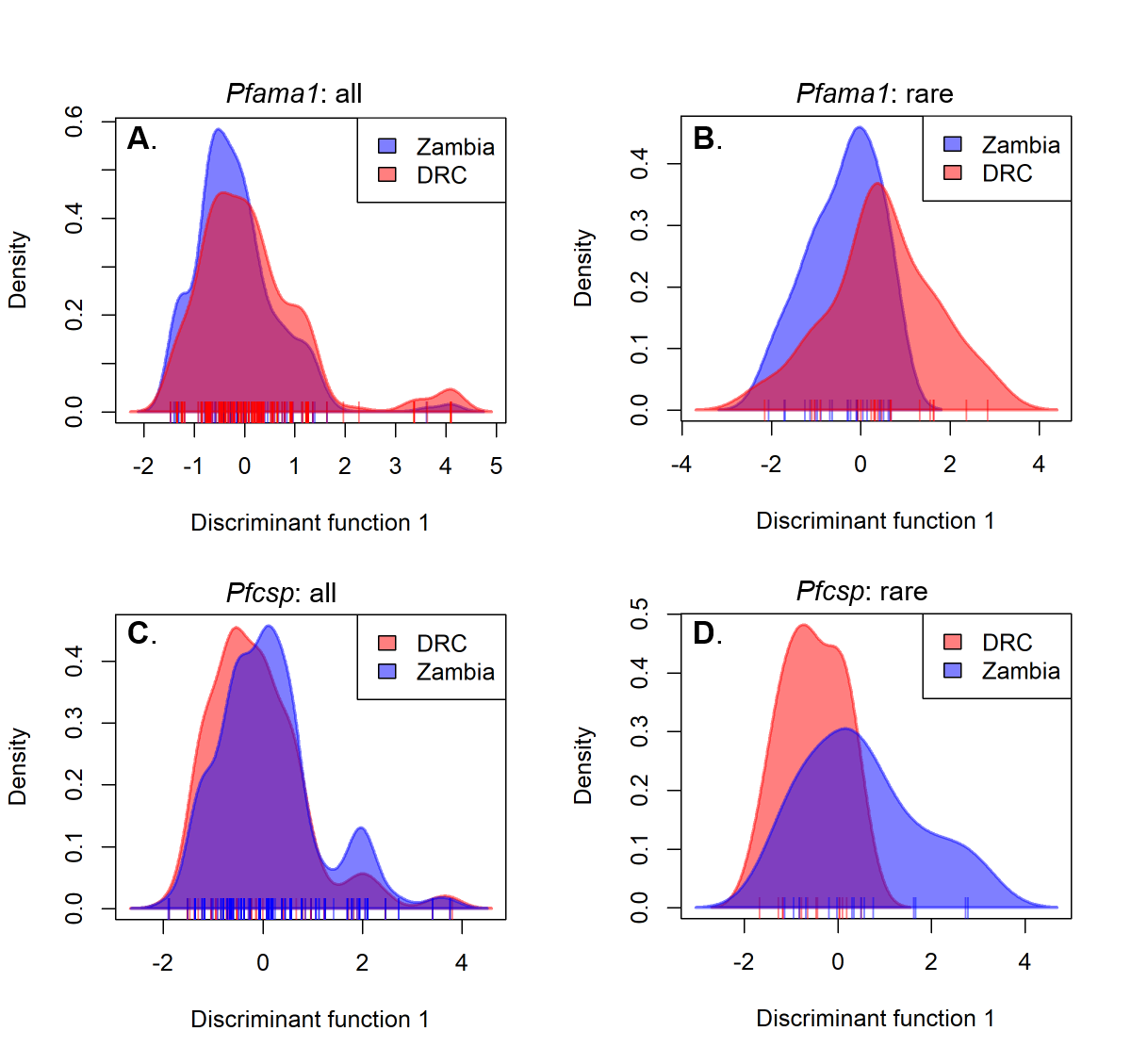
**

**Figure S6:** Dicriminatory analysis of principal components (DAPC) was performed using R package, adgenet. DAPC performs linear discriminat analysis on principal components in order to maximize separation of *a priori* groups. A,B: *Pfama1*; C,D: *Pfcsp*. A,C: DAPC performed using all sequences regardless of population frequency shows no linear function that can classify the principal components of the parasite seqeunces reliably by country. B,D: DAPC using only rare haplotypes (singletons) results in more refined population discrimination for *Pfcsp*.

**Figure S7:**

**
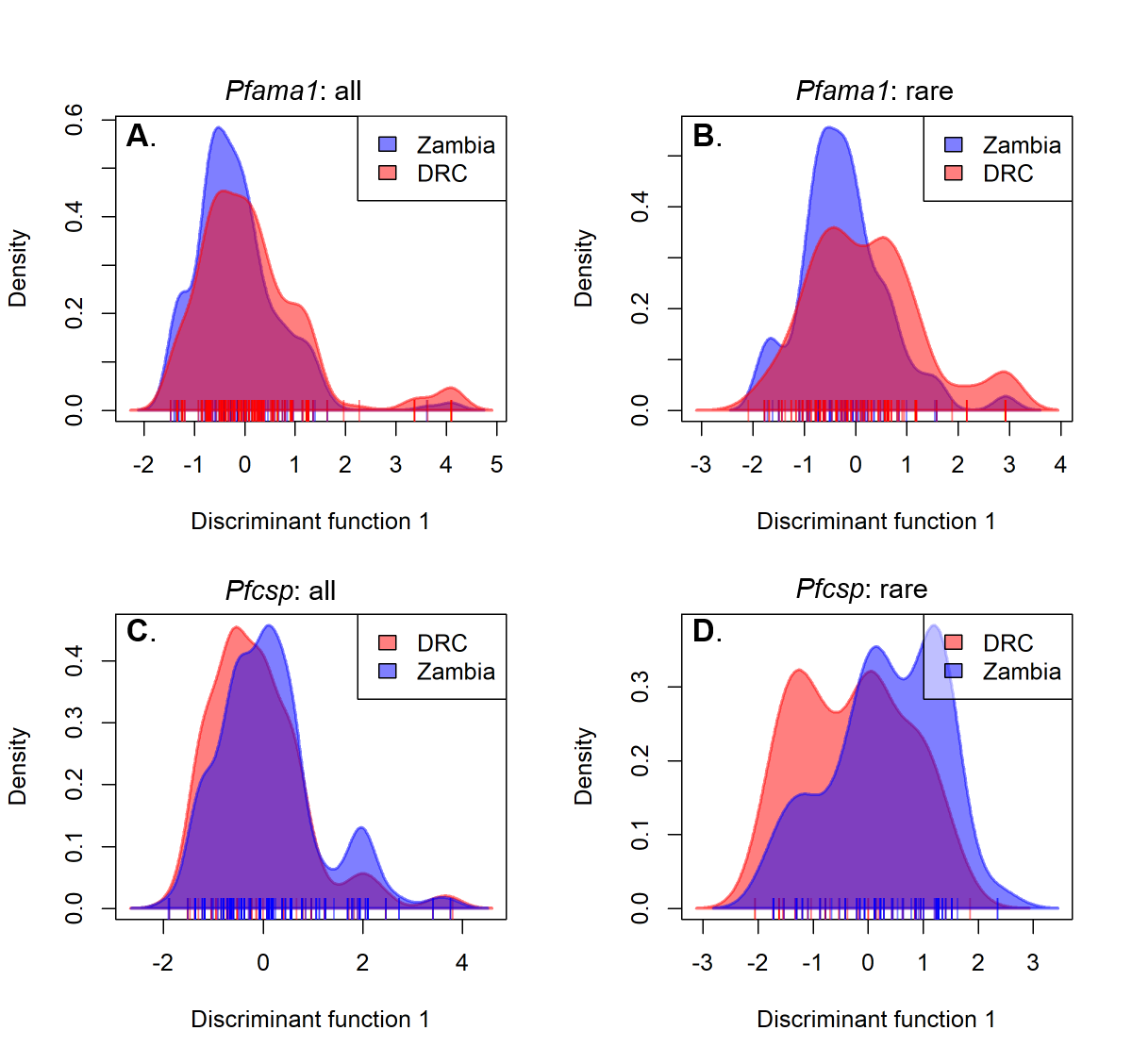
**

**Figure S7:** Dicriminatory analysis of principal components (DAPC) was performed using R package, adgenet. DAPC performs linear discriminat analysis on principal components in order to maximize separation of *a priori* groups. A,B: *Pfama1*; C,D: *Pfcsp*. A,C: DAPC performed using all sequences regardless of population frequency shows no linear function that can classify the principal components of the parasite seqeunces reliably by country. B,D: DAPC using only rare haplotypes (2% or less frequency) results in more refined population discrimination for *Pfcsp*.
